# Effects of Acepromazine-Xylazine-Ketamine combination on physiological and clinical parameters in somali goats (*Capra hircus*)

**DOI:** 10.1186/s12917-026-05783-3

**Published:** 2026-08-06

**Authors:** Abdirahman Saed Abdi

**Affiliations:** Department of Planning and Research, Ministry of Livestock and Rural Development, Hargeisa, Somaliland

**Keywords:** Acepromazine, Field Anesthesia, Ketamine, Somali goat, Somaliland, TIVA, Xylazine

## Abstract

**Background:**

In Somaliland, veterinary surgical interventions are often constrained by the lack of inhalation anesthesia. Total Intravenous Anesthesia (TIVA) offers a portable alternative. This study evaluated the physiological and clinical effects of an Acepromazine (ACP), Xylazine (XY), and Ketamine (KT) protocol in Somali goats, a breed adapted to arid environments.

**Methods:**

Twelve healthy Somali goats (9 females, 3 males; 15–25 kg) were used in a prospective study. Animals were premedicated with Xylazine (0.1 mg/kg) and Acepromazine (0.05 mg/kg) intravenously (IV), followed 10 min later by Ketamine (4 mg/kg) IV. Heart rate (HR), respiratory rate (RR), and rectal temperature (RT) were monitored at 10-minute intervals. Clinical parameters (pedal, corneal, jaw, and tongue reflexes) were assessed for duration of suppression.

**Results:**

HR and RT showed gradual, non-significant declines (*P* > 0.05). RR exhibited significant variation (*P* = 0.034), with a transient increase at 10 and 20 min. Duration of pedal reflex suppression was 20.7 ± 0.7 min. Ptyalism (hypersalivation) was observed in 75% of subjects, and labored breathing in 50%.

**Conclusions:**

The ACP-XY-KT combination provides approximately 20 min of surgical recumbency. While cardiovascular trends remained stable, ptyalism and respiratory shifts necessitate vigilant airway management.

## Background

Goats (*Capra hircus*) are indispensable to the socio-economic fabric of Somaliland, serving as a primary source of protein, milk, and export revenue. As veterinary services expand in the region, the demand for humane surgical interventions—ranging from wound debridement and fracture stabilization to emergency laparotomies—has increased significantly. However, the lack of specialized anesthetic infrastructure, particularly inhalation anesthesia machines and compressed oxygen, remains a major barrier to modern veterinary practice in East Africa [1, 2].

In resource-limited settings, “balanced” protocols utilize low-dose combinations to capitalize on synergistic effects [3]. The combination of Xylazine (α2-adrenoceptor agonist), Acepromazine (phenothiazine), and Ketamine (dissociative) is hypothesized to provide improved sedation and muscle relaxation. While Xylazine and Ketamine provide some analgesia, this combination is primarily intended to facilitate short procedures; invasive surgeries would likely require supplemental local blocks [4].

While the effects of these drugs are well-documented in temperate goat breeds, there is a significant knowledge gap regarding their impact on the Somali goat. Local breeds in arid regions often exhibit distinct metabolic rates and physiological responses to pharmacological agents due to environmental adaptation [5]. Understanding the “anesthetic window” and the physiological shifts in this specific breed is essential for establishing safe clinical guidelines for local practitioners.

The objective of this study was to evaluate the clinical efficacy and physiological safety of an ACP-XY-KT protocol in Somali goats. We hypothesized that this combination would provide a stable plane of anesthesia suitable for short-duration surgical procedures with manageable side effects.

## Methods

### Ethical approval and animal welfare

This study was conducted at the Veterinary Teaching Hospital of the University of Hargeisa. The experimental protocol was reviewed and approved by the Research and Ethics Review Committee of the University of Hargeisa (Ref No: UoH/CVM/RERC/03/2025). All animal handling procedures adhered to the international guidelines for the care and use of laboratory animals.

### Study animals and environmental conditions

Twelve adult Somali goats (9 females, 3 males), aged 1.8 to 2.5 years and weighing between 15 and 25 kg, were selected for the study. The animals were already owned by the University of Hargeisa and were sourced from the university’s teaching farm. Animals were confirmed healthy (ASA Grade I) based on physical examination, packed cell volume (PCV), and fecal analysis. The goats were housed in a semi-intensive system and acclimatized to the hospital environment for 14 days. Prior to the study, goats were fasted for 12 h to prevent ruminal bloat, though water was provided *ad libitum* until 6 h before the procedure.

### Anesthetic protocol

All animals were administered a premedication mixture consisting of 0.1 mg/kg Xylazine (Xyla, Interchemie, Netherlands) and 0.05 mg/kg Acepromazine (Acepromazine 1%, Vetoquinol, France). This combination was delivered as a single intravenous (IV) bolus through the left jugular vein. Exactly 10 min following premedication, anesthesia was induced using Ketamine HCl (Ketamine 10%, Interchemie, Netherlands) at a dosage of 4 mg/kg, administered as a rapid IV bolus. Immediately following the loss of consciousness, all subjects were positioned in right lateral recumbency on a padded surface to prevent pressure-related injury and to facilitate monitoring.

### Monitoring and data collection

HR was monitored via auscultation; RR via thoracic excursions; and RT via digital thermometer. Reflexes were scored as 0 (absent) or 1 (present). The pedal reflex was tested by firm digital pressure on the interdigital space. The corneal reflex was tested by lightly touching the cornea with a sterile cotton swab. No surgical procedures were performed; reflex suppression was used as a proxy for anesthetic depth.

### Statistical analysis

Statistical analysis was performed using IBM SPSS Statistics (Version 23). Data normality was assessed using the Shapiro-Wilk test. A One-way Repeated Measures ANOVA was employed to determine the effect of time on physiological parameters. Post-hoc comparisons were made using the Bonferroni test. Data are presented as Mean ±Standard Error of the Mean (SEM), and *P* ≤ 0.05 was considered statistically significant.

## Results

### Physiological parameters

HR declined from 101.5 ± 2.1 bpm to 84.1 ± 6.2 bpm (*P* > 0.05) (Table 1). Similarly, the Rt decreased slightly throughout the monitoring period, though this trend did not reach statistical significance (Table 1). In contrast, the Rr exhibited significant temporal variation (*P* = 0.034). Rr increased at T10 (34.1 ± 1.8 breaths/min) before declining to 19.5 ± 0.9 breaths/min at T50.

**Table 1:** Effects of ACP-XY-KT on physiological parameters in Somali goats (Mean ± SEM, *N* = 12)

Physiological Parameters	0 min	10 min	20 min	30 min	40 min	50 min
Hr (bpm)	101.5 ± 2.1	86.4 ± 1.2	88.2 ± 2.4	87.5 ± 4.1	86.8 ± 4.8	84.1 ± 6.2
Rr (breaths/min)	26.9 ± 2.2	34.1 ± 1.8*	33.4 ± 2.5*	24.8 ± 2.1	25.5 ± 3.8	19.5 ± 0.9
Rt (°C)	37.6 ± 0.4	37.3 ± 0.2	37.4 ± 0.3	37.2 ± 0.3	36.7 ± 0.3	36.8 ± 0.2

Values are mean ± Standard error of mean, *Indicates significant difference from baseline (*P* < 0.05)

### Reflex suppression and anesthetic depth

The protocol induced rapid unconsciousness in all subjects. The mean duration of surgical anesthesia, as measured by the loss of the pedal reflex, was 20.7 ± 0.7 min. Muscle relaxation was particularly profound, with the tongue reflex remaining suppressed for an average of 23.9 ± 1.8 min (Table 2). (Fig. 1).

**Table 2:** The effect of anesthetic protocol on selected reflexes (*N* = 12)

Reflex	Mean Duration (min)	Standard Error (± SEM)
Jaw Reflex (Jr)	21.8	0.9
Pedal Reflex (Pr)	20.7	0.7
Corneal Reflex (Cr)	20.9	1.1
Tongue Reflex (Tr)	23.9	1.8

Values are mean (min) ± Standard error of mean of four reflexes

**Fig. 1:**
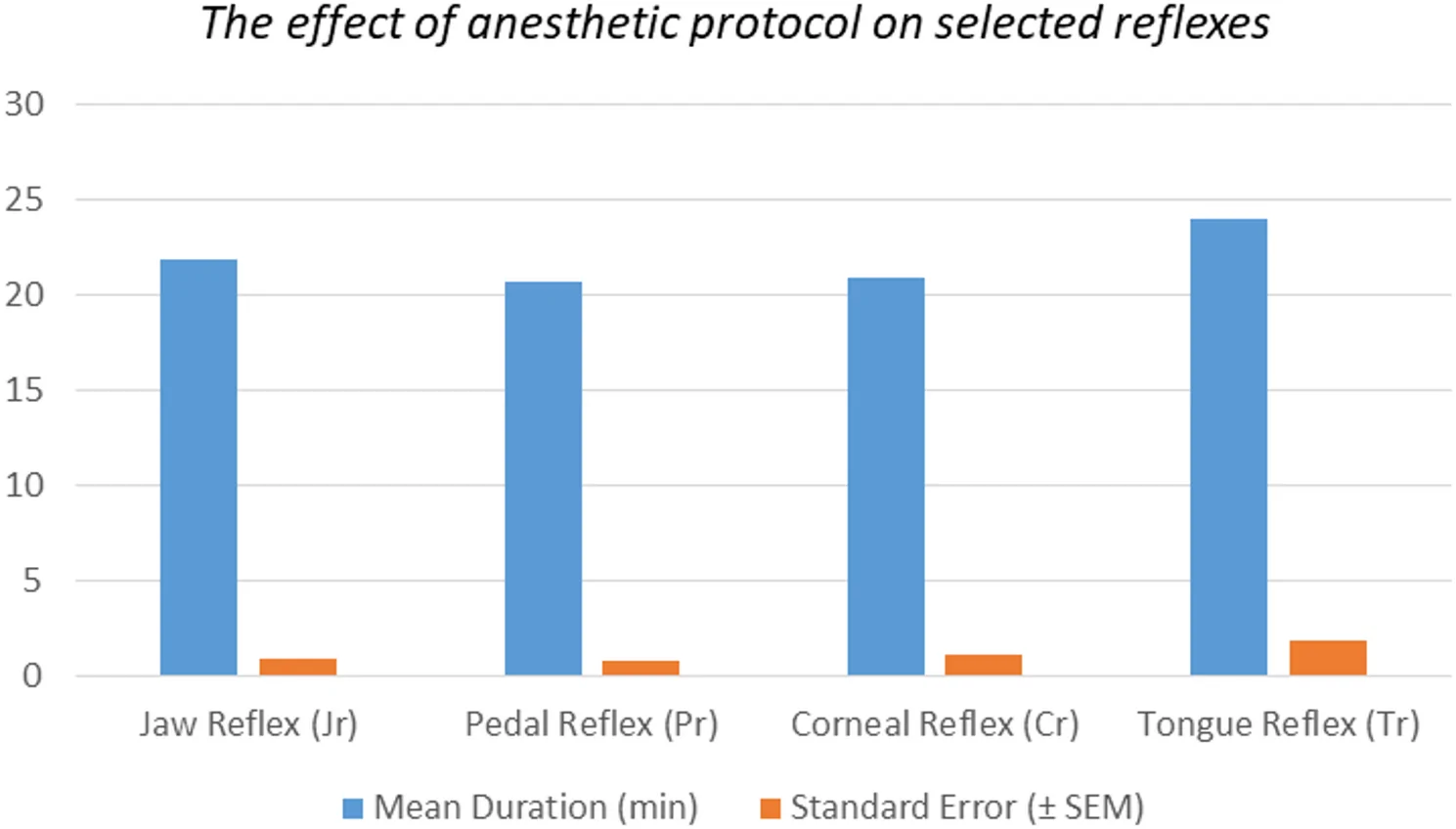
The effect of anesthetic protocol on selected reflexes

### Adverse effects and recovery

Ptyalism (sialorrhea) occurred in 75% of goats. Labored breathing and grunting (expiratory sound) were noted in 50% during the 20–30 min interval. Mean time to sternal recumbency (recovery) was 42 ± 5 min, with transient ataxia (staggering) noted in 50% of subjects (Table 3). (Fig. 2).

**Table 3:** Summary of the side effects observed in experimental goats (*N* = 12)

Side effects	Presence in Animals (*n* = 12)	Percentage (%)
Salivation	9	+ 75%
Labored Breathing	6	+ 50%
Snoring	0	-100%
Grunting	6	+ 50%
Tail wagging	0	-100%
Paddling movement	0	-100%
Staggering	6	+ 50%
Open eyelid	3	+ 25%

Absent, + present, % of goat affected

**Fig. 2:**
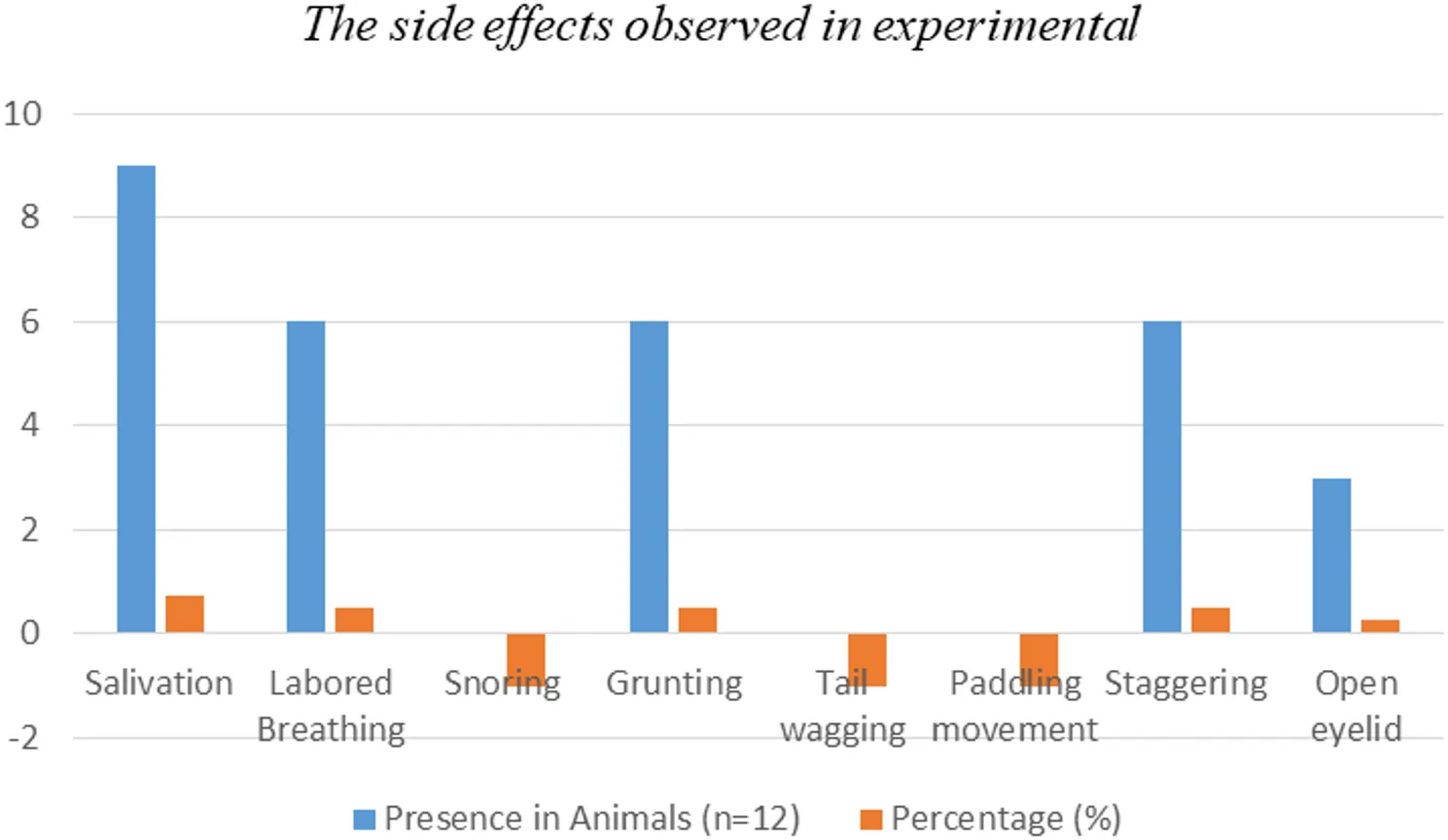
The side effects observed in experimental

## Discussion

The protocol provided stable cardiovascular trends. The mild bradycardia is likely due to the combined effects of Xylazine-induced decrease in sympathetic outflow and increased vagal tone [6, 7]. We acknowledge that “hemodynamic stability” cannot be fully confirmed without blood pressure monitoring.

The observed tachypnea at T10 followed by labored breathing suggests that while ACP does not cause significant muscle relaxation, the synergistic effect of XY and KT may impact upper airway patency in lateral recumbency [8, 9]. Positioning the head with the poll elevated is critical to manage ptyalism and prevent the risk of aspiration pneumonia [10].

This study lacked a control group (e.g., XY-KT without ACP), making it difficult to isolate the specific contribution of Acepromazine. Furthermore, the higher proportion of females (9:3) may not account for potential sexual dimorphism in drug metabolism. No pharmacological antagonists (e.g., Atipamezole) were used, which could have shortened the ataxic recovery phase.

## Conclusion

The ACP-XY-KT protocol is a viable field option for Somali goats, providing 20 min of recumbency. Practitioners must prioritize airway positioning to mitigate the risks of ptyalism and respiratory depression.

## Data Availability

The datasets generated and analyzed during the current study are available from the corresponding author on reasonable request.

## Abbreviations

ACP: Acepromazine
ANOVA: Analysis of Variance
ASA: American Society of Anesthesiologists
Bpm: Beats per minute
Cr: Corneal reflex
Hr: Heart rate
IV: Intravenous
Jr: Jaw reflex
KT: Ketamine
PCV: Packed Cell Volume
Pr: Pedal reflex
Rr: Respiratory rate
Rt: Rectal temperature
SEM: Standard error of the mean
TIVA: Total intravenous anesthesia
Tr: Tongue reflex
XY: Xylazine
